# Predictive Value of Heart Rate Variability for Postoperative Atrial Fibrillation in Off-Pump Coronary Artery Bypass Patients

**DOI:** 10.3390/medicina61060984

**Published:** 2025-05-26

**Authors:** Juš Kšela, Jan Kafol, Viktor Avbelj, Jurij Matija Kališnik

**Affiliations:** 1 Department of Cardiovascular Surgery, University Medical Centre Ljubljana, 1000 Ljubljana, Slovenia; 2 Faculty of Medicine, University of Ljubljana, 1000 Ljubljana, Slovenia; jan.kafol@kclj.si (J.K.); jurij.kalisnik@kabeg.at (J.M.K.); 3 Department of Vascular Diseases, Division of Internal Medicine, University Medical Centre Ljubljana, 1000 Ljubljana, Slovenia; 4 Department of Communication Systems, Jožef Stefan Institute, 1000 Ljubljana, Slovenia; viktor.avbelj@ijs.si; 5 Department of Cardiothoracic and Vascular Surgery, University of Graz affiliated Clinic KABEG, 9020 Klagenfurt, Austria

**Keywords:** atrial fibrillation, heart rate variability, DFA Alpha 1, coronary artery bypass grafting

## Abstract

*Background and Objectives:* Postoperative atrial fibrillation (AF) is a frequent complication after coronary artery bypass grafting (CABG), and is particularly associated with poor outcomes. Heart rate variability (HRV), a non-invasive marker of autonomic function, has been proposed as a tool to predict AF risk, but its utility in off-pump CABG remains unclear. This study aimed to evaluate the predictive value of preoperative HRV parameters, including nonlinear metrics, for postoperative AF in patients undergoing off-pump CABG. *Materials and Methods*: We prospectively enrolled 67 patients undergoing elective off-pump CABG. HRV was assessed using 15 min high-resolution ECGs. Linear and nonlinear HRV parameters were analyzed. Postoperative AF was monitored through continuous ECG (days 0–4), daily 12-lead ECGs (days 5–7), and a 24 h Holter ECG on day 7. Statistical comparisons between AF and non-AF groups were performed, and the predictive accuracy was evaluated using ROC analysis. *Results*: Postoperative AF occurred in 40.3% (n = 27) of patients. Standard HRV measures (total power, frequency components, LF/HF ratio) did not differ significantly between groups. However, preoperative DFA Alpha 1 was significantly lower in patients who developed AF (*p* = 0.010) and showed the highest predictive value (AUC = 0.725, specificity = 80%). Alpha 1 also remained significantly reduced postoperatively in the AF group. Other nonlinear parameters, such as low and average fractal dimension, were also lower postoperatively in the AF group. *Conclusions*: Traditional HRV parameters showed limited predictive value for postoperative AF following off-pump CABG. The nonlinear DFA Alpha 1 index demonstrated a moderate predictive performance and may serve as a useful marker of autonomic dysregulation. Incorporating nonlinear HRV measures into preoperative assessment may improve AF risk stratification.

## 1. Introduction

Postoperative atrial fibrillation (AF) is a common complication following coronary artery bypass grafting (CABG), occurring in up to 34% of patients [1]. Its development has been consistently associated with an increased short- and long-term risk of adverse outcomes, including stroke, thromboembolism, and heart failure [2,3]. Several risk factors have been identified for the development of postoperative AF. Patient-related factors include advanced age, hypertension, obesity, diabetes mellitus, and chronic obstructive pulmonary disease. Intraoperative and postoperative factors, such as the use of cardiopulmonary bypass, electrolyte imbalances, and systemic inflammation, also contribute to the risk. Despite advances in surgical techniques and perioperative care, POAF remains a significant clinical challenge [4]. Detecting postoperative AF is crucial for enabling long-term rhythm surveillance, which may help identify patients at risk of recurrent AF episodes. Management strategies typically involve rate control, with rhythm control considered for symptomatic patients [5]. However, the benefit of anticoagulation therapy in this context is uncertain [6].

Heart rate variability (HRV) is a non-invasive measure of autonomic nervous system function, reflecting the balance between sympathetic and parasympathetic activity [7]. While traditional HRV analyses focus on time and frequency domains, nonlinear methods—such as detrended fluctuation analysis (DFA) and fractal dimension (FD)—offer insights into the complexity and self-similarity of heart rate dynamics over time [8]. HRV has been widely applied to assess cardiac autonomic regulation, with reduced HRV often indicating impaired autonomic function and being associated with various cardiovascular conditions, including hypertension, heart failure, and myocardial infarction [9,10]. Specifically, diminished HRV has been linked to an increased risk of AF, as it may reflect autonomic imbalances that predispose individuals to arrhythmias [11]. It has been demonstrated that lower preoperative HRV correlates with a higher incidence of postoperative AF following CABG, suggesting its potential utility in identifying patients at an elevated risk of this complication [12]. 

Although previous studies have linked reduced HRV with an increased risk of postoperative AF following CABG, the findings remain inconsistent, and the predictive utility of specific HRV parameters is still debated [12,13]. Importantly, data are especially limited for patients undergoing off-pump CABG procedures, where autonomic dynamics may differ from conventional surgery [14]. Off-pump CABG avoids the use of cardiopulmonary bypass, thereby reducing the systemic inflammatory response and neurohumoral stress associated with on-pump procedures [15]. This difference in physiological impact may lead to distinct patterns of autonomic nervous system activity postoperatively, potentially influencing the incidence of atrial fibrillation. Understanding these variations is crucial for improving risk stratification and tailoring perioperative management strategies.

Our study aims to address this gap by evaluating the predictive value of preoperative HRV parameters for postoperative AF in this specific surgical population, with the goal of improving risk stratification and guiding preventive strategies.

## 2. Materials and Methods

This study was approved by the National Medical Ethics Committee of Slovenia and conducted in accordance with the Declaration of Helsinki. All patients were informed about the purpose and procedures of the study, including potential inconveniences and safety measures during the assessments. Written informed consent was obtained from all participants prior to their enrollment in the study.

### 2.1. Study Population and Enrollment Criteria

A total of 67 patients admitted for elective off-pump CABG at the University Clinical Department of the Medical University of Warsaw, Poland, were enrolled in the study. The inclusion criteria comprised adult male and female patients scheduled for isolated off-pump CABG surgery due to stable two- or three-vessel coronary artery disease, with a documented sinus rhythm based on a 24 h Holter ECG recording prior to surgery, and on chronic β-blocker therapy.

Patients were excluded if they had concomitant valvular heart disease; any preoperative rhythm other than sinus, including conduction abnormalities; documented episodes of AF, atrial flutter, atrial tachycardia, or non-sustained ventricular tachycardia lasting more than 30 s; clinically significant bradyarrhythmias lasting over 30 s; the presence of a temporary or permanent pacemaker; preoperative treatment with antiarrhythmic drugs other than β-blockers; diabetes with advanced neurological complications; end-stage renal disease on hemodialysis; thyroid disorders; prior stroke; myocardial infarction within one month before surgery or early postoperative infarction; the need for prolonged (>48 h) postoperative intubation, inotropic support, or intra-aortic balloon pump; the inability to continue β-blocker therapy within 48 h after surgery; or conversion to cardiopulmonary bypass during surgery.

### 2.2. Arrhythmia Surveillance

Preoperative arrhythmias were excluded via the analysis of 24 h Holter ECG recordings. Following surgery, patients were transferred to the intensive care unit, where continuous ECG monitoring with automatic arrhythmia detection (Welch Allyn Propaq CS, Welch Allyn Inc., Skaneateles Falls, NY, USA) was performed during the first four postoperative days. From postoperative day five to seven, patients were clinically assessed daily using 12-lead ECG recordings daily. A second 24 h Holter ECG was performed on postoperative day seven. In case of suspected arrhythmia or clinical deterioration, additional monitoring was initiated. Arrhythmias were defined as atrial fibrillation/flutter or atrial tachycardia lasting ≥ 30 s, clinically relevant bradyarrhythmias ≥ 30 s, progression from simple to complex ventricular ectopy (Lown I–II to III–V), or (non)sustained ventricular tachycardia or fibrillation.

Preoperative β-blocker therapy was resumed postoperatively as soon as patients were hemodynamically stable, and in all cases within 48 h following surgery. Although the exact timing and dosage adjustments were individualized based on clinical judgment, patients were generally restarted on their preoperative regimen.

### 2.3. ECG Measurement Protocol

High-resolution 24 h Holter recordings were conducted one day before and on the seventh day after surgery using 3-channel digital recorders (Aspel AsPEKT 702, 128 Hz, Zabierzów, Poland; Schiller MT-101, 125 Hz, Baar, Switzerland). Short-term (15-min) high-resolution ECGs were recorded preoperatively and on postoperative days three and seven using a 1-channel digital ECG device (DEKG, Intekom, Ljubljana, Slovenia, 1000 Hz), always between 14:30 and 18:00 and at least two hours postprandially in a quiet, temperature-controlled room. Recordings were generally performed around 15:00–16:00, but minor variations within this window occurred due to the clinical workflow. Patients rested supine for 10 min before recordings. A modified CM5 lead configuration was used. For iHRT assessment, five premature ventricular contractions were synchronously induced via temporary epicardial pacing wires unless frequent spontaneous ventricular premature contractions were already present. All recordings were stored and transferred for offline analysis. Patients were advised to abstain from smoking, alcohol, and caffeine for 24 h prior to ECG measurements.

### 2.4. HRV Analysis

All ECG data were analyzed from previously stored recordings. The RR intervals from 15 min ECG recordings were identified using HolCard 24W software version 6.33 (Aspel, Zabierzów, Poland), which applies signal interpolation to enhance the temporal resolution to 1 ms. Automated beat classification (normal, ventricular/supraventricular premature, artifacts) was manually reviewed and corrected.

Linear heart rate variability (HRV) parameters—including the total power (TP), low-frequency (LF) power, high-frequency (HF) power, and LF:HF ratio—were calculated from 15 min high-resolution recordings using HolCard 24W (Aspel, Poland). These linear parameters quantify the magnitude of variability in heart rate intervals, with the LF and HF power reflecting the influence of sympathetic and parasympathetic branches of the autonomic nervous system, while the LF/HF ratio provides an estimate of sympathovagal balance [16].

For nonlinear analyses, cleaned normal-to-normal (NN) RR interval series underwent additional filtering to correct abnormal intervals. Detrended fluctuation analysis (DFA) was used to determine short-term (α1) and long-term (α2) scaling exponents with freely available software from PhysioNet: Detrended Fluctuation Analysis, version 1.0.0 (available at: https://physionet.org/physiotools/dfa/; accessed on 5 June 2008) [17,18]. DFA Alpha 1 quantifies the fractal scaling properties of heart rate dynamics over short intervals, with lower values indicating reduced complexity and autonomic adaptability [8]. Fractal dimension (FD) was computed using Higuchi’s algorithm with software developed by Acharya et al. FD assesses the complexity and irregularity of RR interval time series, where lower values reflect diminished signal complexity, potentially indicating autonomic dysfunction [19,20,21]. Multifractal parameters (τ(q = 3), h_top, Δh) were calculated using open-access tools provided by PhysioNet: Software for Analysis of Multifractal Time Series version 1.0.0 (available at: https://physionet.org/physiotools/multifractal/; accessed on 5 June 2008) [22].

### 2.5. Statistical Analysis

Data were collected using Excel 365 (Microsoft Corporation, Redmond, WA, USA) and analyzed in R version 4.4.1 (R Foundation for Statistical Computing, Vienna, Austria). Patients with missing values for a given variable were excluded from its respective analysis. The number of excluded patients per parameter ranged from none to three, as detailed in Table 1. Distribution normality was assessed using the Shapiro–Wilk test. As most variables were normally distributed, continuous data are reported as means with standard deviations (SDs), and comparisons between patients with and without AF were conducted using either the unpaired *t*-test or Wilcoxon rank-sum test, as appropriate. For within-group comparisons of pre- and postoperative values, the paired *t*-test or Wilcoxon signed-rank test was applied, depending on the distribution. Differences in postoperative changes (delta values) between the AF and No AF groups were assessed using either the unpaired *t*-test or Mann–Whitney U test. *p*-values were adjusted for multiple testing using the Benjamini–Hochberg procedure to control the false discovery rate. Receiver operating characteristic (ROC) analysis was used to evaluate the predictive performance of the preoperative parameters for postoperative AF. For each parameter, the area under the curve (AUC), optimal cutoff value (based on Youden’s index), sensitivity, specificity, positive predictive value, and negative predictive value were calculated. All statistical tests were two-tailed, and *p*-values < 0.05 were considered statistically significant.

## 3. Results

Patients who developed postoperative AF were significantly older than those who did not, with a mean age of 70.3 ± 7.4 years in the AF group compared to 58.9 ± 8.6 years in the No AF group (*p* < 0.001). The detailed clinical and perioperative characteristics of the study population are presented in Table 2.

**Figure 1:**
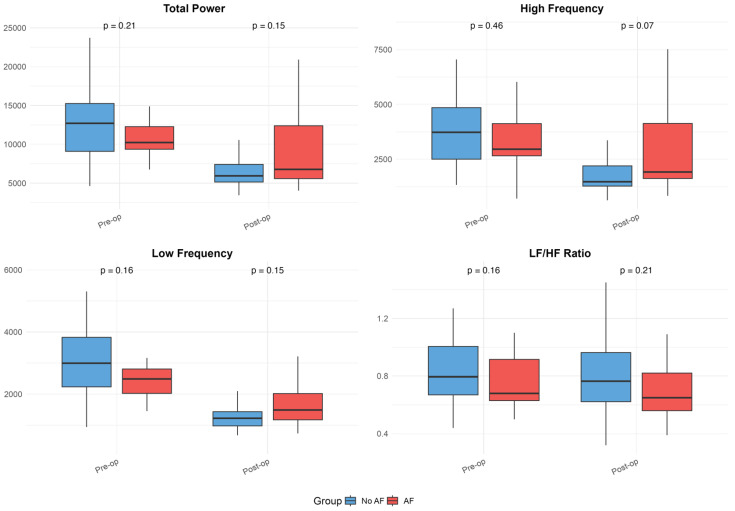
Heart rate variability (HRV) parameters in patients with and without postoperative atrial fibrillation. Boxplots illustrate preoperative and postoperative values of HRV metrics (Total Power, High-Frequency Power, Low-Frequency Power, and LF/HF Ratio) in patients who developed atrial fibrillation and those whose sinus rhythm remained. These plots visually represent the data summarized in Table 1, emphasizing group differences, variability, and trends over time. Boxplots show medians, interquartile ranges (IQR), and whiskers extending to 1.5 times the IQR; outliers were excluded for improved readability.

**Figure 2:**
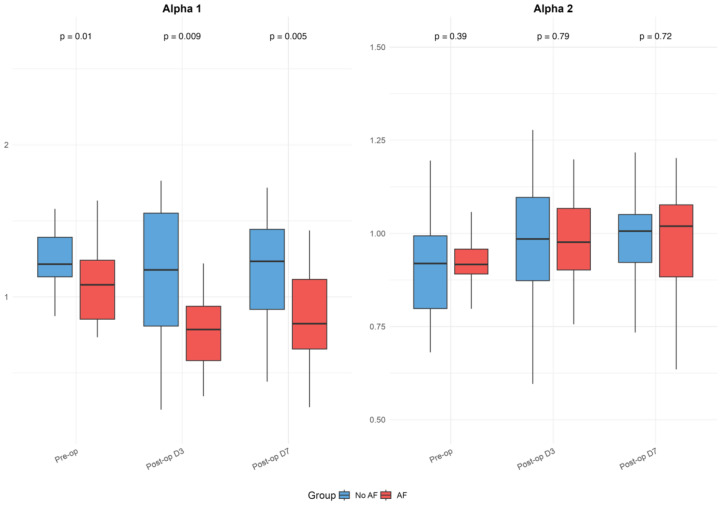
Detrended fluctuation analysis (DFA) Alpha parameters in patients with and without postoperative atrial fibrillation. Boxplots display Alpha 1 and Alpha 2 values before surgery and on postoperative days 3 and 7, as detailed in Table 1. The figure highlights differences between groups and temporal patterns in DFA parameters. Boxplots show medians, interquartile ranges (IQR), and whiskers extending to 1.5 times the IQR; outliers were excluded for clarity.

**Figure 3:**
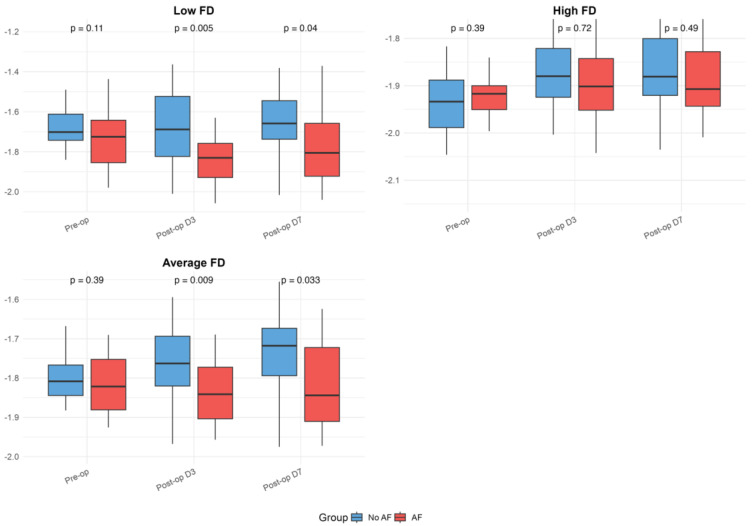
Fractal dimension (FD) parameters in patients with and without postoperative atrial fibrillation. Boxplots present preoperative and postoperative values of Low, High, and Average FD in both groups. The figure serves as a graphical representation of the data shown in Table 1, allowing a visualization of the trends and variability. Boxplots show medians, interquartile ranges (IQRs), and whiskers extending to 1.5 times the IQR; outliers were excluded for improved readability.

The baseline and postoperative values of HRV, FD, and DFA alpha parameters are presented in Table 1 and illustrated in Figure 1, Figure 2 and Figure 3. While no significant differences were observed in standard HRV indices such as the total power, low and high-frequency power, or LF/HF ratio, group differences emerged in more complex signal features. Specifically, Alpha 1 values were significantly lower in the AF group both preoperatively (*p* = 0.010) and on postoperative days 3 (*p* = 0.009) and 7 (*p* = 0.005). Similarly, the AF group showed significantly more negative values of low FD on postoperative day 3 (*p* = 0.005) and day 7 (*p* = 0.040), as well as an average FD on day 3 (*p* = 0.009) and day 7 (*p* = 0.033), indicating altered autonomic regulation and reduced signal complexity in those who developed AF.

Within-group analyses showed significant postoperative reductions in total power, as well as low- and high-frequency components across the overall cohort (all *p* < 0.001). These changes remained significant in the No AF group (all *p* < 0.001), while the decrease in total power did not reach significance in the AF group (*p* = 0.064). Alpha 1 values decreased significantly postoperatively in the overall group (day 3: *p* = 0.005; day 7: *p* = 0.028) and in patients with AF (day 3: *p* = 0.001; day 7: *p* = 0.014), but not in those without AF. Alpha 2 increased after surgery in the overall group (day 3: *p* = 0.007; day 7: *p* = 0.001) and the No AF group (day 3: *p* = 0.033; day 7: *p* = 0.001), with no significant change in the AF group. High FD values significantly decreased postoperatively in both subgroups (No AF: *p* ≤ 0.001; AF: *p* = 0.053). The reductions in low and average FD were more pronounced in the AF group (LFD day 3: *p* = 0.017; AFD day 7: *p* = 0.892), though were not always significant in the overall or No AF groups. No significant within-group changes were observed for the LF/HF ratio. Although the delta values indicated greater postoperative shifts in the AF group for several parameters, the between-group differences in these changes were not statistically significant after FDR correction.

**Figure 4:**
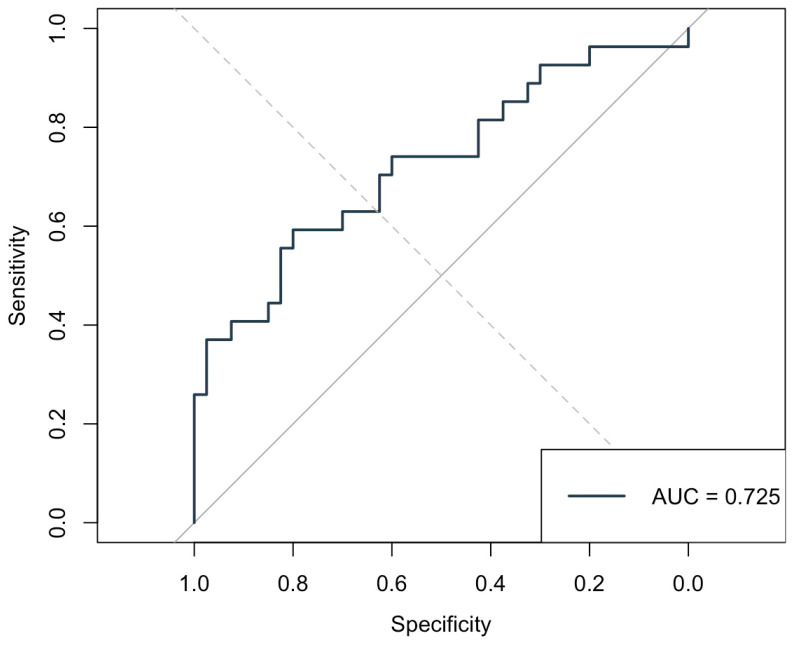
Receiver Operating Characteristic (ROC) curve for preoperative DFA Alpha 1 predicting postoperative atrial fibrillation. The Area Under the Curve (AUC) was 0.725, indicating good discriminative ability.

Among the preoperative predictors of AF, the DFA Alpha 1 parameter demonstrated the highest discriminative performance, with an AUC of 0.725 (see Figure 4). At an optimal cutoff of 1.116, Alpha 1 achieved a sensitivity of 59.3% and specificity of 80%, with a positive predictive value of 66.7% and negative predictive value of 74.4%. This suggests that lower Alpha 1 values preoperatively are moderately effective in identifying patients at risk of developing AF, balancing false positives and negatives better than traditional HRV indices such as total power (AUC = 0.607), high frequency (AUC = 0.564), and low frequency (AUC = 0.625). Parameters like LF0 and the LF/HF ratio (AUC = 0.624) also showed modest predictive value, but Alpha 1 outperformed them in both specificity and overall capacity for discrimination, highlighting its potential as a better marker of autonomic dysregulation related to postoperative AF. Clinically, a preoperative Alpha 1 value below 1.116 could serve as a threshold to identify patients at an increased risk of postoperative AF, supporting its potential use in preoperative risk stratification and prompting the consideration of enhanced perioperative rhythm monitoring or preventive strategies.

## 4. Discussion

This study evaluated the predictive value of preoperative HRV for postoperative AF in off-pump CABG patients. We found that traditional HRV measures had limited predictive value, while lower preoperative Alpha 1 values were significantly associated with postoperative AF, highlighting its potential as a risk marker.

Standard and frequency-domain ratios in our cohort showed minimal differences between those who developed AF and those who remained in sinus rhythm, suggesting that baseline autonomic tone captured by linear metrics may not strongly discriminate AF risk. This aligns with prior observations that traditional HRV measures often perform only slightly better than chance in this setting [14]. However, some studies have reported stronger associations between preoperative linear HRV measures and postoperative AF. For instance, Kinoshita et al. found that higher 24-h Standard deviation of NN intervals (SDNN) values were associated with increased AF risk, with SDNN achieving fair predictive accuracy [23]. Similarly, Thanh et al. observed that very low SDNN modestly predicted AF up to six months postoperatively [12]. These findings contrast with our results and may reflect methodological differences, including the use of 24-h recordings instead of short-term HRV, on-pump versus off-pump surgery, and longer AF surveillance windows.

DFA α1 emerged as the most promising HRV parameter in our study, with a moderate discriminative ability (AUC 0.725) for predicting postoperative AF. Our results align with and extend prior evidence supporting the value of nonlinear HRV metrics in identifying patients at risk for postoperative AF. The short-term fractal scaling exponent α1, which reflects correlations in heart rate fluctuations over short intervals [24], was significantly lower in patients who developed AF both before and after surgery—suggesting that subtle breakdowns in autonomic regulation may predispose to arrhythmogenesis even before surgical insult. The pronounced reductions in Alpha 1 and fractal dimension in the AF group likely reflect heightened sympathetic activation and impaired parasympathetic modulation in response to surgical stress and inflammation, even in off-pump CABG. These autonomic imbalances may contribute to atrial electrical instability, promoting arrhythmogenesis through enhanced ectopic activity and reentry mechanisms [25]. Importantly, the identified preoperative DFA Alpha 1 cutoff of 1.116, although not sufficient as a standalone diagnostic marker, could serve as a practical threshold for risk stratification. Patients with Alpha 1 values below this level might be considered at higher risk of developing postoperative AF and could benefit from more intensive perioperative rhythm monitoring or tailored preventive strategies, such as early β-blocker optimization or prophylactic antiarrhythmic therapy. However, the clinical application of this cutoff should be interpreted with caution and requires validation in larger, prospective cohorts before it can be recommended for routine use.

Our findings are consistent with those of Tarkiainen et al. and Wu et al., both of whom reported reduced DFA α1 in patients who developed AF after CABG [26,27]. Wu et al. further demonstrated that ischemic preconditioning mitigated the reduction in α1, reinforcing its role as a marker of autonomic adaptability [27]. More recently, Kališnik et al. demonstrated that reduced DFA Alpha 1, combined with the PR interval, independently predicted postoperative AF in a larger and more homogeneous on-pump cardiac surgery cohort, achieving an AUC of 0.804. The higher predictive accuracy observed in their study, compared to our off-pump setting (AUC 0.725), may reflect the more uniform autonomic response to cardiopulmonary bypass and lower variability in DFA Alpha 1 measurements (SD 0.17 vs. 0.25 in our study) [28]. Nevertheless, these complementary findings from diverse surgical settings further support DFA Alpha 1 as a robust marker of autonomic dysregulation and arrhythmia risk. Our study builds on these insights by applying standardized, high-resolution recordings in an off-pump CABG cohort—a population where HRV-based prediction has been particularly variable. Unlike earlier studies by Hakala et al. and Chamchad et al., which found minimal predictive value in standard HRV measures, our results suggest that nonlinear indices such as DFA α1 may overcome these limitations [13,14]. Moreover, a recent systematic review by Matusik et al. highlighted DFA α1 as a promising tool for perioperative risk stratification, emphasizing its ability to capture the deviations in autonomic balance not reflected in traditional HRV metrics [25]. Our findings support this conclusion, underlining DFA α1’s potential as a valuable component in multimodal preoperative assessment strategies.

From a practical perspective, DFA Alpha 1 could potentially be integrated into existing preoperative risk models alongside established clinical predictors such as age, atrial size, or prior arrhythmias. Its inclusion might help refine perioperative risk stratification by identifying patients who would benefit from closer rhythm monitoring or preventive therapies. Nevertheless, the feasibility of routine high-resolution ECG acquisition and nonlinear HRV analysis in clinical settings remains uncertain. Before such an approach can be adopted in standard practice, further studies are needed to evaluate the cost-effectiveness, workflow integration, and reproducibility of DFA Alpha 1 measurements across diverse populations and clinical environments.

This study has several limitations. The relatively small sample size, determined based on feasibility without a formal power calculation, may have reduced the statistical power and limited our ability to detect subtle differences in HRV parameters, particularly among traditional metrics. All participants were white, which may affect the study’s generalizability to more diverse populations. Autonomic regulation and HRV patterns may differ across ethnic groups, potentially influencing both baseline HRV values and susceptibility to postoperative AF. Additionally, variations in surgical protocols, perioperative management, and healthcare systems across centers or regions might further affect the generalizability of our findings. In addition, patients with diabetes with neurological complications and thyroid disorders were excluded to minimize confounding, as these conditions are known to significantly affect autonomic function and HRV measurements [29,30,31]. However, their exclusion limits the applicability of our findings to the broader CABG population, in which such comorbidities are common. Although β-blocker therapy was resumed within 48 h in all patients, individual variability in timing and dosage adjustments—data which were not systematically recorded—may have influenced the HRV values, particularly on postoperative day 3. Postoperative monitoring was limited to seven days, so late-onset AF episodes could have been missed. Additionally, the single-center design may not reflect outcomes in other settings. Minor variabilities in the ECG recording time within the afternoon window may have introduced confounding due to circadian influences on HRV. Finally, while some HRV parameters showed predictive value, their clinical utility remains to be confirmed in larger, multi-center studies.

Taken together, our study adds to the growing body of evidence showing that certain HRV metrics—particularly those capturing nonlinear dynamics—may offer valuable predictive information for postoperative AF. However, their overall performance remains moderate, warranting cautious interpretation. These findings suggest that HRV should be integrated with other clinical predictors or biomarkers to enhance risk stratification, rather than be used in isolation for AF prediction following CABG.

## 5. Conclusions

This study demonstrated that while traditional HRV parameters were limited in predicting postoperative AF after off-pump CABG, the nonlinear DFA Alpha 1 index showed moderate discriminative ability. Lower preoperative Alpha 1 values were consistently associated with AF, suggesting its potential as a marker of autonomic dysregulation. Although not suitable as a standalone tool, Alpha 1 may enhance preoperative risk assessment when integrated with other clinical predictors and should be further validated in larger cohorts.

## Figures and Tables

**Table 1:** Comparison of preoperative and postoperative HRV parameters in patients with and without postoperative atrial fibrillation.

Parameter	Overall (N = 67)	No AF (N = 40)	AF (N = 27)	*p*-Value
Total Power pre-op (ms^2^)	13,333.0 ± 9760.3	14,601.2 ± 11,892.2	11,381.9 ± 4531.2	0.210
Total Power post-op (ms^2^) ^a^	7864.3 ± 4469.3	7114.6 ± 3997.2	9220.9 ± 5036.6	0.147
High Frequency pre-op (ms^2^)	3967.7 ± 2570.8	4219.7 ± 2907.6	3580.2 ± 1933.3	0.459
High Frequency post-op (ms^2^) ^a^	2250.3 ± 1539.1	1944.1 ± 1317.5	2804.3 ± 1777.0	0.065
Low Frequency pre-op (ms^2^)	3269.5 ± 3664.9	3689.1 ± 4592.8	2624.0 ± 1140.4	0.156
Low Frequency post-op (ms^2^) ^a^	1564.2 ± 1024.8	1466.7 ± 1101.4	1740.6 ± 866.3	0.147
LF/HF Ratio pre-op	0.82 ± 0.24	0.85 ± 0.25	0.78 ± 0.23	0.156
LF/HF Ratio post-op ^a^	0.77 ± 0.26	0.80 ± 0.26	0.72 ± 0.26	0.210
Alpha 1 pre-op	1.17 ± 0.25	1.25 ± 0.18	1.05 ± 0.29	0.010
Alpha 1 post-op day 3 ^b^	1.01 ± 0.42	1.13 ± 0.44	0.8 ± 0.29	0.009
Alpha 1 post-op day 7 ^c^	1.05 ± 0.38	1.18 ± 0.37	0.86 ± 0.32	0.005
Alpha 2 pre-op	0.92 ± 0.11	0.91 ± 0.13	0.93 ± 0.08	0.394
Alpha 2 post-op day 3 ^b^	0.98 ± 0.16	0.97 ± 0.18	0.98 ± 0.12	0.793
Alpha 2 post-op day 7 ^c^	0.99 ± 0.14	1.0 ± 0.14	0.98 ± 0.16	0.719
Low FD pre-op	−1.71 ± 0.11	−1.68 ± 0.09	−1.74 ± 0.13	0.108
Low FD post-op day 3 ^b^	−1.74 ± 0.19	−1.68 ± 0.19	−1.84 ± 0.14	0.005
Low FD post-op day 7 ^c^	−1.72 ± 0.19	−1.67 ± 0.18	−1.79 ± 0.18	0.040
High FD pre-op	−1.93 ± 0.06	−1.93 ± 0.07	−1.92 ± 0.05	0.394
High FD post-op day 3 ^b^	−1.88 ± 0.09	−1.87 ± 0.09	−1.88 ± 0.09	0.719
High FD post-op day 7 ^c^	−1.87 ± 0.09	−1.86 ± 0.09	−1.88 ± 0.08	0.489
Average FD pre-op	−1.81 ± 0.06	−1.8 ± 0.06	−1.82 ± 0.07	0.394
Average FD post-op day 3 ^b^	−1.79 ± 0.1	−1.76 ± 0.1	−1.83 ± 0.08	0.009
Average FD post-op day 7 ^c^	−1.77 ± 0.11	−1.74 ± 0.1	−1.82 ± 0.11	0.033

Data are presented as mean ± standard deviation. Statistical comparisons were performed using the paired *t*-test or Wilcoxon signed-rank test, depending on the data distribution. To control the false discovery rate, *p*-values were adjusted using the Benjamini–Hochberg method, with significance set at an adjusted *p* < 0.05. Legend: AF = atrial fibrillation; pre-op = preoperative (prior to coronary artery bypass grafting). Footnotes: ^a^ Missing 1 value; ^b^ Missing 3 values; ^c^ Missing 2 values.

**Table 2:** Clinical, and perioperative characteristics of the study population (N = 67).

Variable	Value
Coronary artery disease	
Two-vessel disease	21 (31%)
Three-vessel disease	46 (69%)
Prior myocardial infarction	33 (49%)
Arterial hypertension	60 (90%)
Diabetes mellitus	19 (28%)
Dyslipidemia	67 (100%)
Left ventricular ejection fraction (%)	55.3 ± 9.8
Left atrial diameter (cm)	4.0 ± 0.5
EuroSCORE	2.9 ± 2.6
Number of grafts performed	2.5 ± 0.9
Medication use (admission/discharge)	
β-blockers	100%/100%
ACE inhibitors	53%/51%
Calcium channel blockers	13%/2%

## Data Availability

Data is available from the corresponding author upon reasonable request.
